# Principles of risk assessment in colon cancer: immunity is key

**DOI:** 10.1080/2162402X.2024.2347441

**Published:** 2024-04-30

**Authors:** Assia Hijazi, Jérôme Galon

**Affiliations:** a INSERM, Laboratory of Integrative Cancer Immunology, Paris, France; b Equipe Labellisée Ligue Contre le Cancer, Paris, France; c Centre de Recherche des Cordeliers, Sorbonne Université, Université Paris Cité, Paris, France; d Veracyte, Marseille, France

**Keywords:** Immunoscore (IS), digital pathology, stage II colon cancer (CC), biomarkers, risk assessment, high risk, tumor recurrence, adjuvant chemotherapy (ACT), risk factor, survival benefit

## Abstract

In clinical practice, the administration of adjuvant chemotherapy (ACT) following tumor surgical resection raises a critical dilemma for stage II colon cancer (CC) patients. The prognostic features used to identify high-risk CC patients rely on the pathological assessment of tumor cells. Currently, these factors are considered for stratifying patients who may benefit from ACT at early CC stages. However, the extent to which these factors predict clinical outcomes (*i.e*. recurrence, survival) remains highly controversial, also uncertainty persists regarding patients’ response to treatment, necessitating further investigation. Therefore, an imperious need is to explore novel biomarkers that can reliably stratify patients at risk, to optimize adjuvant treatment decisions. Recently, we evaluated the prognostic and predictive value of Immunoscore (IS), an immune digital-pathology assay, in stage II CC patients. IS emerged as the sole significant parameter for predicting disease-free survival (DFS) in high-risk patients. Moreover, IS effectively stratified patients who would benefit most from ACT based on their risk of recurrence, thus predicting their outcomes. Notably, our findings revealed that digital IS outperformed the visual quantitative assessment of the immune response conducted by expert pathologists. The latest edition of the WHO classification for digestive tumor has introduced the evaluation of the immune response, as assessed by IS, as desirable and essential diagnostic criterion. This supports the revision of current cancer guidelines and strongly recommends the implementation of IS into clinical practice as a patient stratification tool, to guide CC treatment decisions. This approach may provide appropriate personalized therapeutic decisions that could critically impact early-stage CC patient care.

## Introduction

Stage II colon cancer (CC) represents an early-stage of the disease wherein the tumor has not metastasized to lymph nodes or distant organs. This stage is featured by a high cure rate (approximately 80%) following surgical resection of the primary tumor.^1^ Notably, stage II CC accounts for only 30% of all colon cancers with a relatively low rate of relapse. Traditionally, adjuvant chemotherapy (ACT) has been the standard treatment approach for patients with locally advanced tumors, particularly in stage III CC. The benefits of ACT following tumor resection translate into long-term survival rates ranging between 8.7% and 21.5%, contingent upon the T-and *N*- tumor stage classification.^2^ However, the administration of ACT for stage II patients remains highly controversial, despite numerous clinical trials and meta-analyses.^3^ Previous clinical trials administering ACT with fluoropyrimidines (5-fluorouracil) have only shown marginal benefits in high-risk patients,^4–8^ (Table 1), complicating the approach to these patients and the assessment of the role and efficacy of ACT in the stage II setting.

**Table 1.** Key adjuvant chemotherapy trials in stage II colon cancer.

Trials and meta-analysis	No. of patients	Adjuvant chemotherapy (ACT)	Clinical Outcomes
QUASAR	3,239	5-FU/LV, with or without Lemavisole vs observation	5-y OS improvement: 83.9% vs 81.5%
Ontario Group (pooled data from 12 trials)	4,187	5-FU vs obsevation	DFS increased from 5 vs 10% OS not statistically significant (HR = 0.87; 95%Cl 0.75 to 1.10, *p* = 0.07)
IMPACT (Pooled data from 5 trials)	1,016	5-FU/LV vs observation	No significant improvement in 5y-OS (82% vs 80%)
MOSAIC	2,246	LV5-FU2 vs FOLFOX4	5-y DFS: 73.3% in the FOLFOX4 and 67.4% in LV5FU2 group, (HR = 0.80; 95% CI, 0.68 to 0.93; *p* = .003). No significant improvement in 6-y OS (86.9% vs 86.8%, respectively (HR = 1.00; 95% CI, 0.70 to 1.41; *p* = .986). Adding oxaliplatin to LV5FU2 significantly improved 5-year DFS and 6-y OS
TOSCA	1,254	FOLFOX (5-FU, leucovorin, and oxaliplatin) or CAPOX (capecitabine plus oxaliplatin) for *3 months* vs *6 months*	5-y DFS was 82.2% for the 3-month arm vs 88.2% for the 6-month arm, (HR = 1.41;95% CI, 1.05–1.89; *p* = .86 for non inferiority). With CAPOX: 5-y DFS almost similar in the 2 arms (difference = 0.76%) (95% CI, −6.28% to 7.80%). With FOLFOX: 5-y DFS is more beneficial (8.56%) in 6-month (95% CI, 3.45%-13.67%)
SCOT	6,088	Oxaliplatin and 5-fluorouracil vs oxaliplatin and capecitabine, administered over 3 or 6 months.	3-y DFS in the 3-month (76.7%) vs 6-month (77.1%); HR = 1.006 (p-value for non-inferiority = 0.012).
ACHIEVE-2	514	patients (255 in the 3-month arm; 259 in the 6-month arm) were treated with 5-FU, LV and oxaliplatin (mFOLFOX6) vs capecitabine combined with oxaliplatin (CAPOX).	With CAPOX: 3-y DFS was not significant between 3-month arm (88.2%) and 6-month arm (88.4%) (HR = 1.13; 95% CI, 0.65–1.96).

DFS: Disease Free Survival; OS: Overall Survival; 5-FU: 5-Fluorouracil; LV: Leucovorin; ACT: Adjuvant Chemotherapy; HR: hazard ratio.

There exists a significant gap in knowledge regarding the predictive value of tumor features in stage II CC, which would enable the selection of patients most likely to benefit from ACT. Additionally, the correlations between tumor features and the appropriate chemotherapy regimen remain unclear. These issues have sparked debates regarding the patient population that would benefit from adjuvant therapy, as well as the optimal type of chemotherapy (monotherapy *vs* doublet therapy) that must be recommended and the survival endpoint to define.

This clinical situation underscores the urgent need for risk stratification methodologies to discern patients likely to derive significant benefit from post-operative chemotherapy, based on their risk of recurrence.

The classification of Stage II CC patients into high and low-risk groups is not definitely clear.

In clinical practice, patient risk stratification relies on various factors such as tumor size (T), lymph node involvement (N), tumor grading, and the necessity for emergency surgery. Unfortunately, these features lack the precision to consistently predict the risk of recurrence for each individual patient. Moreover, the early definition of high-risk stage II CC appears imperfect, as many patients with identified high-risk features do not experience tumor relapse.^9^

Several multigene assays, including the ColDx assay,^10^ Oncotype Dx^11^ and ColoPrint,^12^ have been previously developed to assess recurrence and classify stage II patients into high and low-risk categories for potential ACT benefits,^10–14^ However, their application in clinical routine has not been recommended due to insufficient evidence supporting their ability to identify patients who would benefit from ACT^15^ (National Comprehensive Cancer Network (NCCN) clinical practice guidelines in oncology. Available at: https://www.nccn.org/professionals/physician_gls/pdf/gist.pdf).

The American Society of Clinical Oncology (ASCO)^16,17^ and the European Society for Medical Oncology (ESMO) guidelines did not advocate for the routine application of ACT in low-risk subgroups of stage II CC patients. This recommendation stems from a meta-analysis indicating that, on average, only 5% of patients who received ACT experienced a 5-year survival benefit.^17^

**Figure 1.**
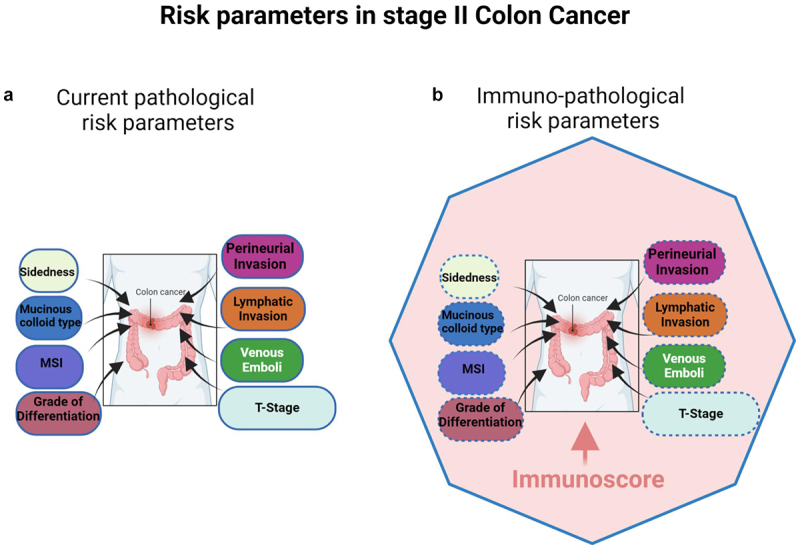
(a) Multivariable analysis of risk parameters in stage II CC patients. The size of the box represents the importance (hazard ratio) of each parameter regarding prognosis, according to current pathological parameters. (b) Multivariable analysis of risk parameters in stage II CC patients. The size of the box represents the importance (hazard ratio) of each parameter regarding prognosis, according to immuno- pathological parameters. Immunoscore (the pink zone) occupies the highest hazard ratio and revealed as the sole significant parameter in multivariable analysis, therefore being the strongest and most significant risk-parameter in stage II CC. The plain blue line of the polygon represents significance in cox multivariable analysis, specifically referring to immunoscore, while the dotted blue lines of the boxes denote non-significance for all the cited risk parameters.

In clinical settings, ACT is exclusively recommended for patients with stage II CC carrying high-risk features, despite the absence of significant benefit from randomized studies. According to ASCO and the National Comprehensive Cancer Network® (NCCN) reports, the current guidelines outline various pathological high-risk parameters, including one or more of the following prognostic indicators: T4 stage (being the grade of tumor invasion into the bowel wall), poorly differentiated histology (G3), deficient mismatch repair/microsatellite instability (dMMR/MSI), lympho-vascular and perineural invasion, emergency clinical presentation (bowel obstruction or perforation/Venous emboli), tumor budding and inadequate lymph node (LN) sampling (<12 nodes), Mucinous colloid type, sidedness^18^ (Figure 1(a)). For patients with 1–7 retrieved LNs, the 5y-OS rate was 49.8%, increasing to 56.2% with 8–12 LNs, and further to 63.4% with > 13 LNs.^18^ Currently, the main risk factors considered include T4 stage, lymph node number, grading, and microsatellite status. Stages IIB and IIC patients with T4 tumors, or those with lesions penetrating to the visceral peritoneum or invading the surrounding organ, are classified at higher risk of recurrence and are recommended ACT administration.^16^ Conversely, stage IIA patients with other high-risk factors, including sampling of fewer than 12 lymph nodes, vascular emboli, lymphatic invasion, and perineural invasion (VELIPI), poorly or undifferentiated tumor grade, intestinal obstruction, tumor perforation, or grade BD3 tumor budding, may optionally receive ACT.^16^ Furthermore, it has not been recommended for patients with mismatch repair deficiency/microsatellite instability (dMMR/MSI) tumors to be offered ACT. However, in case of combination of dMMR/MSI with high-risk factors, oxaliplatin-containing chemotherapy is recommended.^16^ For patients eligible for adjuvant doublet chemotherapy, adjuvant oxaliplatin-containing chemotherapy may be administered for either 3- or 6- months.^19^

The co-existence of multiple risk factors may increase the risk of recurrence.^20^ For example, the 5-year disease-free survival (DFS) rate stood at 74.8% for stage II patients presenting two or more risk factors, compared to 87.3% for those with only one risk factor.^19^ Numerous clinical trials (Table 1), observational reports and retrospective analyses have investigated the role of ACT in high-risk stage II patients.^6,21–24^ Subgroup analyses were conducted on stage II CC patients who received FOLFOX as ACT.^25^ Among the 899 patients diagnosed with stage II CC, 330 were classified as having low-risk disease and 569 as having high-risk disease. However, in high-risk patients, the application of FOLFOX did not yield statistically significant improvements in either DFS or overall survival (OS).^25^

Further exploratory analyses on high-risk patients revealed that fluorouracil-based ACT could only improve survival by less than 5%,^26^ exposing patients to the adverse effects of chemotherapy. Given the heterogeneity within stage II CC, accurate risk assessment and patient stratification are crucial factors in guiding adjuvant treatment decisions.^27^

Several subgroup analyses of adjuvant trials have demonstrated that stage II disease with one or more prognostic features presents a relative long-term DFS benefit from ACT, similar to that observed in stage III patients^22,28–30^ (Mosaic Trial, Table 1). Nevertheless, unlike stage III, the unexpected lack of surrogacy of DFS for OS in stage II may be attributed to differences in disease biology.^31^ The disparities observed among existing studies (Absence *of ACT benefits vs relative benefits*) may derive from variations in the baseline risks of the enrolled patient population. This has prompted the need to refine stratification strategies to accurately assess risks and predict treatment outcomes in individual stage II patients. This entails exploring improved predictive markers to identify patients who stand to benefit most from ACT.

We have recently demonstrated the robustness of the consensus Immunoscore (IS) assay in accurately stratifying stage II CC patients into IS-high and -low risk subgroups.^32,33^ Patients within these subgroups have shown significantly divergent clinical outcomes.^32,33^ IS is an immune digital pathology (DP)-based assay that quantifies CD3+ and CD8+ lymphocyte infiltration density in both the tumor core and invasive margin. Across all CC stages (I-III), IS assessment has shown a prognostic value significantly superior to all existing prognostic tumor parameters, including the classical AJCC/UICC TNM staging system^34^ (Figure 1(b)). Further studies have evaluated the potential of IS in predicting response to ACT. In line with earlier research,^34–37^ our recent studies have consistently identified a correlation between a high IS and improved clinical outcomes in all stage II patients.^32,33^ Particularly, among stage II patients with microsatellite stability (MSS), a high IS was associated with improved DFS and reduced risk of recurrence. Notably, in high-risk stage II patients, IS accurately predicted recurrence risk with High IS exhibiting a remarkably similar recurrence risk to that of low-risk stage II patients. This risk was significantly lower than that observed in high-risk stage II patients with Low IS.^32^ The high-risk stage II patients classified solely on histopathological features have shown different clinical outcomes. Consequently, the performance of IS contributes to improved patient stratification,^32^ potentially guiding clinicians toward optimal treatment options for individual patients. Moreover, alongside IS, analysis of other immune cell subpopulations such as CD4 Th1 macrophages provides important additional information pertaining to patient survival and response to therapy.^38–40^ Biomarkers related to specific genetic mutations or alterations within the tumor microenvironment (TME), such as KRAS, BRAF mutations, or microsatellite instability (MSI), hold prognostic significance, thus influencing treatment decisions.^41^ For instance, patients with MSI-high tumors may respond favorably to immune checkpoint inhibitors.^42^ Furthermore, biomarkers associated with inflammatory pathways or TME components, such as cytokines (TNF-α, IL-6, etc…), chemokines (CXCR1, CXCR2, CXCR4, CXCL8, CXCL12, etc.), NF-κB activation, and Cancer Associated-Fibroblasts (CAF) activation may offer prognostic insights and guide treatment strategies.^43–45^ Moreover, tumor budding, a prognostic factor in stage II CRC, characterized by the presence of small clusters of undifferentiated tumor cells at the invasive front, is associated with aggressive tumor behavior and poor prognosis in colorectal cancer.^46,47^ These biomarkers along with many others play critical roles in guiding treatment decisions and predicting clinical outcomes for patients with colorectal cancer.

This review mainly delves into the critical aspects of risk assessment in stage II CC patients, with a particular emphasis on the significance of the IS biomarker in early-stage CC. It highlights the potential of IS as a robust DP tool for stratifying patients into different risk subgroups and discusses its impact on predicting clinical outcomes and guiding ACT decision-making.

### The potential of the consensus IS in predicting survival and risk of relapse in early-stage colon cancer

Recently, the consensus IS has been evaluated in the tumors from two cohorts of 1885 AJCC/UICC-TNM stage I/II CC patients from Canada/USA (cohort 1) and Europe/Asia (cohort 2).^32^ In these patients, immunity emerged as key determinant, surpassing all cancer cell-associated parameters (like grade of differentiation, T4 stage, venous emboli, lymphatic invasion, or perineural invasion (VELIPI)) in predicting outcomes (Figure 1(b)). Among Stage II patients (including Stage II, MSS Stage II, untreated Stage II, high-risk Stage II, and T4 tumors), the immune contexture measured by the IS was the most significant contributor to assess the DFS rate (72%) surpassing all other clinical parameters.^32^

This study reports on the consensus IS as a powerful stratifier for stage II CC patients. The IS reliably diagnoses low immune cell infiltrated patients at risk of relapse, who would therefore benefit from ACT. Conversely, high-IS exhibited the lowest risk of recurrence across both cohorts comprising 1885 patients.

It has been also shown that in stages I/II, 5-year recurrence-free rates were 78.4% (95%-CI, 74.4–82.6), 88.1% (95%-CI, 85.7–90.4), 93.4% (95%-CI, 91.1–95.8) in low, intermediate, and high-IS, respectively (HR (Hi vs. Lo) = 0.27 (95%-CI, 0.18–0.41); *p* < 0.0001). Remarkably, the IS has emerged as the most potent predictor of survival among all other parameters, contributing to 60% of the predictive value. Thereby, IS was found significantly associated with survival in Stage II, high-risk Stage II, T4N0, and MSS patients. Furthermore, patients with high IS had prolonged time to recurrence (TTR) in T4N0 tumors even among those who did not receive chemotherapy. Cox multivariable analysis has revealed the IS as the sole remaining significant parameter for predicting DFS in high-risk stage II patients.^32^ Conversely, patient gender, T-stage, VELIPI status, MSI status, sidedness, or differentiation grade did not emerge as significant prognostic indicators in the same analyses.^32^

Notably, the association of IS with outcomes was independent (TTR: HR (Hi vs. Lo) = 0.29, (95%-CI, 0.17–0.50); *p* < 0.0001) of the T-stage, microsatellite instability-status (MSI), sidedness, and patient gender, as demonstrated in Cox multivariable analysis.^32,33^

This study provided critical evidence indicating that patients with high-pathological risk and a low IS face the highest risk of recurrence, suggesting potential benefits from ACT for this subgroup. Conversely, it was demonstrated that high-pathological risk patients with a high IS exhibit a recurrence risk comparable to that of low-pathological risk patients and could potentially avoid ACT and its associated toxicity.^32^

Overall, these findings demonstrate the critical prognostic value of the IS in high-risk stage II CC and highly recommend the implementation of IS as a patient stratification assay into clinical practice to guide treatment decisions and enhance personalized care in early CC stages.

### Prognostic and predictive value of IS and its association with ctDNA in stage II colorectal cancer

Circulating tumor DNA (ctDNA) is a recently identified prognostic factor and an emerging cancer biomarker that can guide treatment of stage II CC by avoiding the administration of unnecessary ACT without compromising recurrence-free survival.^48^ Among ctDNA-positive patients who received adjuvant chemotherapy, a three-year recurrence-free survival rate of 86.4% was observed.^48^ However, despite the availability of multiple ctDNA assays, recent developments have raised concerns regarding their efficacy and reliability. For instance, the Guardant Health company recently discontinued its cancer blood test in the COBRA trial and halted patient enrollment prematurely. This decision followed an interim analysis that revealed concerns about the test’s specificity, the study’s risky design, and the use of an older generation of minimal residual disease (MRD) assay. (*https://www.medtechdive.com/news/guardant-abandons-cancer-blood-test-trial cobra/692786/)*

Previously, we reported that a high density of CD3+, CD8+ and effector-memory T-cells associated with decreased VELIPI.^49^ Since patients with VELIPI+ are more prone to have detectable ctDNA, we hypothesized a potential relationship between IS and ctDNA detection. To investigate this, we examined the roles of IS and ctDNA in guiding adjuvant treatment in Chinese early-stage colorectal cancer (CRC).^50^ Our recent study demonstrated that IS-High patients, including clinically high-risk individuals, exhibited excellent clinical outcomes with no recurrences observed over a three-year follow-up period. Additionally, the 3-year DFS rates were 100% for IS-High, 76% for IS-Intermediate (IS-Int), and 47% for IS-Low (*p* < 0.001). Notably, IS exhibited strong prognostic value in stage II CRC patients. Multivariate Cox analysis underscored IS as the sole significant parameter associated with DFS, thus proving its clinical utility in prognostication and treatment decision-making.^50^

This study further unveiled that IS-High patients exhibited the lowest rate of positive ctDNA, suggesting that this subgroup may be less likely to benefit from chemotherapy treatments. Intriguinly, among IS-High patients with positive ctDNA who did not receive adjuvant chemotherapy, none experienced relapse during the two-year follow-up period. Additionally, no instances of relapse were observed in IS-High patients, regardless of ctDNA status (ctDNA positive or negative cases). While post-operative ctDNA assay effectively identified patients at heightened risk of recurrence, it was IS that reliably determined patients at low risk of recurrence.^50^ This underscores the complementary roles of ctDNA and IS assessment in guiding treatment decisions and prognostication in early-stage CRC.

Based on these results combining IS with ctDNA assessment, a decision algorithm has been formulated for guiding adjuvant treatment strategies in stage II CC.^50^ Firstly, patients with IS-High had the lowest risk of recurrence and may be spared from ACT. Secondly, patients with negative postoperative ctDNA and an intermediate or low IS demonstrated an intermediate risk of recurrence and therefore could be considered for ACT. Thirdly, patients with positive postoperative ctDNA and either an IS-Int or IS-Low status presented the highest risk of recurrence. Hence, they were strongly recommended for chemotherapy.

### IS digital pathology outperforms expert pathologists’ quantitative visual assessment of immune response

For decades, traditional pathology practices have been instrumental in diagnosis and classification of cancer patients. However, recent landmark innovations in precision medicine have paved the way for the development of DP-based approaches for quantitative analyses. The advent of AI technologies in clinical settings has introduced significant benefits, enabling the exploration and retrieval of high-resolution quantitative information with accuracy and precision that surpasses human visual assessment. Notably, the quantitative evaluation of immune response was not considered a powerful biomarker in clinical pathology until the introduction of IS. This AI-assisted DP-immune assay has revealed the prognostic impact of the immune response in CC patients to improve treatment decision-making in early stages,^32–34,50–52^ In a study comparing immune response assessment in CC using IS (automated DP) and pathologist visual scoring (T-score), four pathologists evaluated tumor specimens from 50 early-stage CC patients and classified CD3+ and CD8+ stained slides.^51^ While digital pathology IS proved highly reproducible,^34^ agreement among pathologists was minimal to weak. Furthermore, a weak concordance between the two methods was observed, resulting in the misclassification of 48% of cases by pathologist scoring. Given the substantial disagreement in interpretation among pathologists, even after training, the IS is unlikely to be reliably reproduced using non-standardized methods.^51^ It is rather highly recommended to use the reproducible and standardized consensus IS using digital pathology.^32–34,52–56^

**Figure 2.**
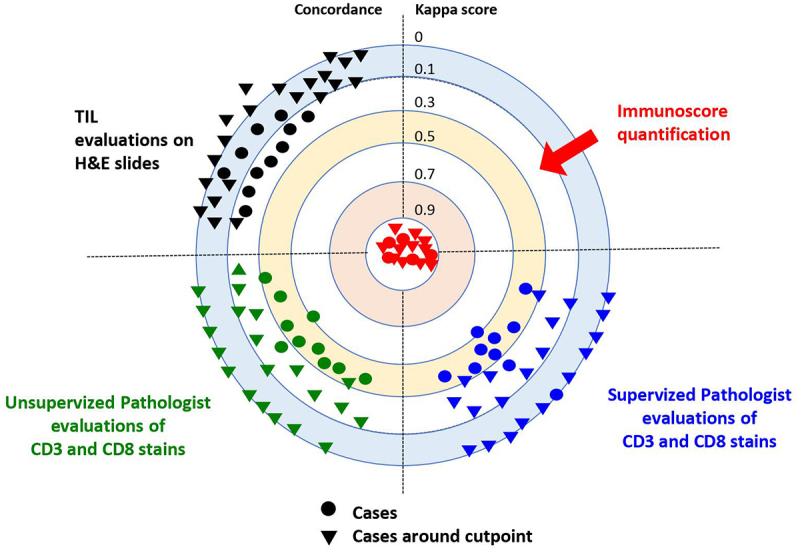
Agreements between pathologists’ T-score and the reference immunoscore are illustrated by Cohen’s kappa scores for 270 colon cancer patients, before and after supervised training. Kappa scores: none (0–0.2), weak (0.4–0.59), moderate (0.6–0.79), strong (0.8–0.9) and almost perfect (>0.9). IS digital pathology quantification showed almost perfect concordance (Cohen’s kappa K > 0.9) in the reproducibility. No concordance (Cohen’s kappa K < 0.25) was observed between TIL evaluated on H&E slides and immunoscore. A weak or minimal concordance (Cohen’s kappa K < 0.5) was observed between pathologists’ visual evaluation of stained CD3 and CD8 slides, both before and after training and with known IS cases. No concordance (Cohen’s kappa K < 0.12) was observed between pathologists’ visual evaluation of stained CD3 and CD8 slides, both before and after training. Each dot represents one observer. Each triangle illustrates evaluation of cases around the cut-points by one observer. Data points and kappa scores are previously described,^52^ supplementary data.

In a recent multi-institutional evaluation study, we compared the performance of the consensus IS to a visual evaluation of the immune response performed by 10 expert pathologists on 540 hematoxylin – eosin (H&E) and CD3+/CD8+ stained slides from 270 randomly selected CC tumors. The IS performed with DP demonstrated high reproducibility and concordance.^52^ However, in more than 92% of cases, pathologists’ T-score evaluations by 10 international expert pathologists were discordant with the IS assay.^52^ Additionally, a disagreement between semi-quantitative visual assessment of T-score and the reference IS was observed in 91% and 96% of cases before and after training, respectively (*Pathologists’ training consisted of providing them with 12 cases of CD3+ and CD8+ stained slides at the cutoff values for IS*). The concordance index between pathologists and the digital IS was significantly weak in two- and three-category IS, according to the statistical analyses. Overall, these results demonstrated that the IS outperformed expert pathologists’ T-score evaluation in the clinical setting^52^ (Figure 2).

**Figure 3.**
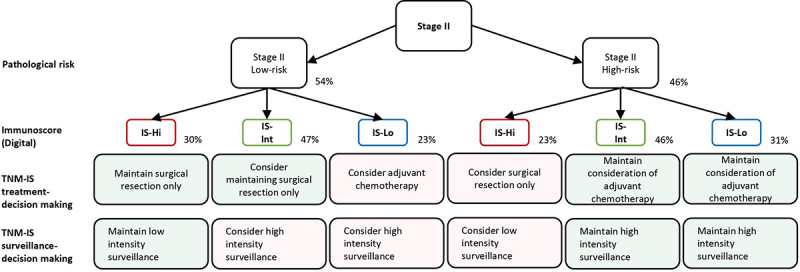
A decision-making algorithm for predicting treatment and surveillance in stage II Low-risk and high risk for IS-Hi, -int and -lo patients’ categories. Immunoscore (IS) could impact treatment decision-making between 23 and 48% of the patients with stage II colon cancer. Immunoscore (IS) could impact surveillance decision-making for 48% of the patients with stage II colon cancer. Visual evaluation of T-score by pathologist would lead to 70% of non concordant cases, leading to inappropriate treatment and surveillance. Percentages derive from clinical decision-tree according to IS in stage II proposed in a prior study,^52^ supplementary data.

Based on our previous treatment decision-making algorithm, IS would impact treatment decision in up to 44% of stage II and 55% of stage III CC patients.^57^ Besides, the non-concordance existing between pathologists’ visual T-scoring and IS would result in the misclassification of 70% of cases^52^ (Figure 3). Consequently, approximately 87.000 CC cases per year would likely be misclassified by pathologist scoring and may receive inadequate treatment.^52^

Therefore, these findings unveiled the potential of the IS as a reproducible assay for the quantitative analysis of tumor-infiltrated immune cells. This immune DP tool can improve diagnosis and patient stratification into prognostic recurrence groups, thereby guiding appropriate therapeutic decisions for CC patients^51,52^ (Figure 3). Furthermore, this research sheds light on the importance of implementing DP in clinical practice for the appropriate personalized treatment of CC patients and potentially other tumor types.

## Discussion

The current literature is widely controversial regarding the classification of stage II CC patients at high risk and their potential benefit from ACT after surgery,^58–60^ There are conflicting results and inappropriate staging guidelines,^61^ making the administration of ACT for stage II CC quite inconclusive. Additionally, the absence of reliable biomarkers predicting response to chemotherapy and the lack of randomized trials demonstrating potential benefits of chemotherapy in high-risk Stage II CC patients significantly complicate treatment decisions in this patient population.

The recent international implementation of the consensus IS assay in stages I/II/III CC^34,62^ has demonstrated the reproducibility of DP and image analysis software. It has also revealed the significant prognostic value of the tumor immune contexture in assessing patients’ immune response and predicting clinical outcomes.^34,39,51,52,63,64^ The consensus IS has proven to be a powerful stratifier for Stage II patients, including Stage II, MSS Stage II, untreated Stage II, high-risk Stage II and T4 tumors.^32^ Moreover, the IS permitted the accurate stratification of CC patients into low- and high-risk subgroups, identifying individuals with good prognostic features who may avoid adjuvant therapy.^32,65^

According to available guidelines, ACT is recommended for patients with high-risk features. However, our findings indicate that a major part of these patients (69.5%), who did not receive ACT but carry an IS-High, present a recurrence risk comparable to that of the low-risk patients.^32^ Additionally, IS serves as a reliable prognostic marker to assess patients’ risk of recurrence. Therefore, IS plays a crucial role in identifying the subgroup of patients at high risk of relapse, guiding the need for critical care monitoring after surgery. Consequently, IS is highly recommended as a test for informing adjuvant treatment decisions in Stage II CC patients.^32^

However, it’s essential to acknowledge the major limitation of this non-randomized study, which stems from its heterogeneous patient population obtained from 13 different countries.^32^ Despite this limitation, our study evaluated the potential of IS in patients from diverse ethnicities and healthcare programs. Moving forward, further efforts are imperative to validate the IS assay in randomized clinical trials and to evaluate its predictive value for response to chemotherapy in these patients. Such endeavors are critical for advancing personalized treatment strategies and optimizing outcomes in Stage II CC patients.

The preexisting immune contexture in cancer patients, could be a driving determinant for different treatment decisions across various stages of the disease.^39,63,64,66–71^ The IS has shown predictive value in identifying patients who benefit from specific treatments, such as 6 months FOLFOX6, within both low- and high-risk pathological stages.^72^ Consequently, IS assessment holds significant clinical relevance in both early- and late-stage CC.^34,73–77^

Moreover, it was lately revealed that stage II CRC patients with IS-Int and IS-Low with the lowest DFS rate could mostly benefit from ACT, while patients with IS-High should not receive ACT.^50^ Thus, IS helps identifying patients with excellent prognostic features, who may be spared from chemotherapy. IS emerged as the most significant biomarker with robust clinical usefulness in adjuvant setting at early-stage CRC.^32,50^ In recent years, IS and postoperative ctDNA have demonstrated superior prognostic value compared to all other clinical parameters in early-stage CRC. Therefore, their implementation into clinical practice is highly recommended to guide adjuvant treatment strategies in early CRC.^50,78^ The preexisting adaptive immunity significantly impact cancer patients’ response to treatments.^74,79,80^ Thus, the misclassification of stage II and III CC patients based on T-score evaluation could lead to improper treatment decision-making.^32,52,72,81–83^ Beside ACT, high-risk patients may also derive benefits from different oncology treatments.^64,66,67,79,84–97^

Thereby, Stage II CC patients identified as high risk based on IS assessment could be misclassified in the low clinical risk-subgroup category.^52^ This leads to inappropriate prediction of their risk of recurrence, resulting in inadequate surveillance for patients considered at high risk of recurrence. This would negatively impact patient health as they may be under-treated, which delays the detection of early symptoms of tumor relapse. Similarly, IS-High stage II CC patients wrongly classified as low T-score by visual examination may be erroneously recommended for ACT, unnecessary exposing them to potential side-effects and toxicity.^52^

As previously discussed, the IS provided highly consistent quantitative analysis of tumor-infiltrated immune cells. This revealed the potential of this immune pathology tool for a better diagnosis and stratification of cancer patients into reliable prognostic groups, based on their immune parameters. According to recent findings, IS quantification of CD3+ and CD8+ cells on a whole digital slide section could avoid potential misclassification of cancer patients, often seen with traditional visual assessment methods.^52^ Therefore, standardized IS assay outperforms visual immune response assessment by expert pathologists in predicting the risk of relapse in stage II CC.^51,52^ These results raise the importance of implementing DP to improve cancer diagnosis and provide appropriate personalized therapeutic decisions that would critically impact patient care management in early-stage CC.^51,52^

7Herein, it is also critical to address the challenge posed by single-point tumor biopsy, which inherently fails to capture the full spectrum of tumor heterogeneity, given the diverse cellular compositions and genetic mutations present at different regions of the tumor. This limitation can result in potential mischaracterization and development of inadequate treatment strategies. To overcome this challenge, novel imaging modalities have emerged as promising alternatives. Techniques such as radiolabelled anti-CD8 imaging and radiomics-based AI provide valuable insights into the immune microenvironment and enable the extraction of quantitative features from medical images, enhancing the ability to comprehensively understand tumor heterogeneity.^98^ These innovative approaches not only could guide clinicians toward more effective and targeted interventions in precision medicine but also highlight the importance of addressing the limitations of traditional biopsy methods. In the realm of combining radiotherapy and immunotherapy, imaging biomarkers and radiomics offer noninvasive tools for assessing the entire disease, including its spatial heterogeneity and temporal changes. Molecular imaging technologies, like positron emission tomography and single-photon emission computed tomography, show promise in tumor detection, predict immunotherapy responses and assess risk parameters.^99^ Also, a radiomic signature can predict disease-free survival in stage II and III CC, serving as a valuable supplement for risk stratification in these patients.^100^ Therefore, the identification of novel imaging biomarkers holds the key to developing personalized treatment plans and addressing challenges in the field of immunotherapy and radio-immunotherapy combinations. Future strategies using these imaging techniques may also contribute to the early stratification of patients, improving the overall management of immunotherapy challenges faced by oncologists. However, it is imperative to acknowledge that the limitations attributed to single-point tumor biopsy are predominantly applicable in the context of Stage IV metastatic cancers and do not extend to early-stage cancers. In such instances, where the primary tumor is surgically excised, pathologists have the opportunity to stain the entire tumor slide. Consequently, IS is performed on the whole tumor slide from the resected primary tumor, with all positive cells meticulously counted via digital pathology. This method yields a comprehensive, objective, and quantitative density of adaptive immune cells within the tumor.^34,39,53–56,101–103^

The prognostic value of IS has been extensively addressed in a variety of cancer types,^39,71,101,104–110^ demonstrating its robust prognostic value regardless of the tumor stage. By stratifying patients according to their tumor immune components, IS can sort individuals at risk and also predict their survival outcomes and treatments. Moreover, the consensus IS has been validated worldwide for its superior prognostic value compared to the classical TNM staging system, even enhancing the prognostication provided by TNM.^34^

For the first time, the immune response criterion has been introduced into the latest (5^th^) edition of the WHO classification of digestive tumors, as *essential and desirable biomarker*. WHO classification of CRC now recommends the inclusion of cytotoxic T-cell densities evaluated in the center and the invasive margin of the tumor, which is performed by the consensus IS. Furthermore, the IS was introduced into the 2020 European ESMO^27^ and into the 2021 Pan-Asian adapted ESMO Clinical Practice Guidelines^111^ to refine prognosis and thus adjust the chemotherapy decision-making process.^27,111^ Given its potent ability to predict cancer recurrence, IS emerged as a novel paradigm for guiding cancer treatment. Additionally, the latest findings may significantly improve risk stratification in stage II CC with the implementation of IS. Thus, integrating IS into clinical practice could help identifying patients who may benefit from ACT.

Overall, this research argues for the revision of the current cancer guidelines (National Comprehensive Cancer Network® (*NCCN*®), College of American Pathologists (CAP), and AJCC/UICC-TNM) to introduce the consensus IS into the clinical prognostic features of cancer patients.

Ultimately, it would be fundamental to implement recent technological advances in AI and the powerful stratification systems into clinical settings to guide clinical decisions and improve treatment outcomes for cancer patients.
